# Characterization of Regulatory T-Cell Markers in CD4+ T Cells of the Upper Airway Mucosa

**DOI:** 10.1371/journal.pone.0148826

**Published:** 2016-02-11

**Authors:** Christina Ballke, Einar Gran, Espen S. Baekkevold, Frode L. Jahnsen

**Affiliations:** 1 Department of Pathology and Centre for Immune Regulation, Oslo University Hospital and University of Oslo, Oslo, Norway; 2 Department of Otorhinolaryngology, Lovisenberg Diakonale Hospital, Oslo, Norway; Jackson Laboratory, UNITED STATES

## Abstract

CD4+ T regulatory cells (Tregs) comprise a heterogeneous population of cells the regulate immune responses and prevent autoimmunity. Most reports on human Tregs are derived from studies of peripheral blood, although Tregs mainly exert their functions in the periphery. Here we performed a detailed analysis of Tregs in the human upper airway mucosa under non-inflammatory conditions, and found that 10% of all CD4+ T cells expressed the transcription factor FOXP3 and the memory marker CD45RO, as well as high levels of CTLA-4. The majority of FOXP3+CD4+ T cells co-expressed the transcription factor Helios and produced very little cytokines, compatible with being thymus-derived Tregs. FOXP3+Helios-CD4+ T cells were more heterogeneous. A mean of 24% produced the immunomodulatory cytokine IL-10, whereas a large fraction also produced IL-2, IFN-μ or IL-17. A significant population (6%) of FOXP3-negative T cells also produced IL-10, usually in combination with IFN-μ. Together, we found that CD4+ T cells in the upper airways differed functionally from their counterparts in peripheral blood, including higher expression of IL-10. Moreover, our findings suggest that several subsets of CD4+ T cells with functionally distinct regulatory properties reside in the upper airway mucosa which should be taken into account when targeting Tregs for therapy.

## Introduction

The environment of the respiratory tract is extremely challenging for the local immune system [1]. In particular, the upper airway mucosa is constantly exposed to a vast variety of harmless, but highly antigenic proteins of plant or animal origin. Maintenance of homeostasis in the airways depends on the ability of the local immune system to be tolerant to such antigens, while at the same time be able to rapidly mount an immune response to microbial pathogens, including a large array of viruses, which use the upper airway mucosa as their primary entry site. Importantly, breakdown of tolerance mechanisms to otherwise harmless antigens may lead to unwanted chronic inflammatory reactions, such as allergic rhinitis, which affects more than 20% of the population in industrialized countries [2].

To maintain homeostasis in the airway mucosa, effector functions of the immune system must be tightly controlled. To accomplish this task, the mucosal immune system has developed several layers of regulatory mechanisms, of which the function of regulatory T cells (Tregs) appears to be particularly important. Tregs are present in most tissues, and studies in experimental mice have shown that they play an important role in inhibiting immune reactions toward environmental antigens encountered at epithelial surfaces [3]. Over the last years it has become evident that T cells with regulatory properties are very heterogeneous, consisting of multiple subsets with distinct origin and functions [4].

The most studied type of Tregs is CD4+ T cells characterized by their expression of the transcription factor FOXP3, which is crucial for their suppressive activity. Most FOXP3+ Tregs are thymus-derived, and have been designated thymus (t) Tregs or naturally occurring (n) Tregs [5]. Such cells have relatively high affinity towards self-antigens [6] and are believed to be particularly important in suppressing autoimmune reactions. Studies of experimental mice have also identified Foxp3+CD4+Tregs that are induced in the periphery, termed peripheral (p)Tregs or inducible (i)Tregs [5]. In several mouse models, pTregs have been shown to be antigen-specific, thus regulating the immune response to foreign antigens [7]. However, tTregs and pTregs display similar phenotypes, and their discrimination in vivo has proven difficult. Recently, however, based on studies in humans and mice it has been suggested that the transcription factor Helios is expressed by tTregs, whereas pTregs are Helios-negative [8,9]. In mice it was furthermore demonstrated that neuropilin-1 was selectively expressed on tTreg cells, and the vast majority of neuropilin-1+ Tregs co-expressed Helios [10], adding to the notion that expression of Helios in Foxp3+CD4+ T cells may be a useful marker to identify tTregs. Recently, it was also shown in experimental mice that Helios was required for stable inhibitory activity of Foxp3+CD4+ Tregs [11]. Moreover, studies in humans have shown that Helios- and Helios+ Treg cells are functionally different. Both express the inhibitory protein CTLA4, but whereas Helios+ Tregs produce little cytokines, Helios- Tregs secrete a variety of cytokines [12–15], including the immunosuppressive cytokine IL-10 [12]. Importantly, the production of IL-10 by Tregs has been shown to play a particularly important role in mucosal tissues of experimental mice [3]. However, the production of IL-10 is not unique for FOXP3+ Tregs as other subsets of T cells also express and secretes this immune-inhibitory cytokine. For example type 1 regulatory (Tr1) cells are FOXP3-negative T cells that develop after birth and produce high levels of IL-10 in response to foreign antigens [16].

Allergic rhinitis is a chronic inflammatory disorder caused by a dysregulated immune reaction to environmental allergens such as pollen and animal dander. Several studies have shown that both allergic rhinitis patients and healthy, non-allergic individuals have allergen-specific Th2 cells, but FOXP3+Tregs and/or Tr1 cells in allergic rhinitis patients are either dysfunctional or reduced in numbers [17,18]. It has also been suggested that immunotherapy changes the composition or increase the number of FOXP3+Tregs or Tr1 cells, and that these changes are partly responsible for the effect of this therapy. Therefore, manipulation of FOXP3+Tregs or Tr1 cells is currently explored as targets of novel treatment strategies to cure allergic rhinitis [19].

Although Tregs primarily function in the tissues in which they reside, most studies of human Tregs have been performed on blood-derived cells. In fact, Tregs are found in most peripheral tissues and it has been shown that selective depletion of FoxP3+ Tregs in the skin of experimental mice results in local inflammation [20]. Moreover, Tregs are influenced by the local tissue environment, and to understand the function of such cells it is necessary to study Tregs in the tissue where they normally reside [21]. Studies of immune cells in peripheral tissues during steady-state are important in order to understand how homeostasis of the local immune system is achieved and maintained. Such knowledge is also crucial in order to understand why immune-regulatory mechanisms break down in various chronic inflammatory reactions such as allergic rhinitis. However, detailed analyses of Tregs in the human airway mucosa have not been reported. To this end, we have performed a detailed characterization of T cells with regulatory properties in the upper airway mucosa under non-inflammatory conditions.

## Materials and Methods

### Subjects

Nasal mucosa prepared for flow cytometry was obtained from the lower edge of the inferior turbinate during surgery for septum deviation. In all cases, the mucosa appeared macroscopically normal and none of the donors had any history of allergic disease (n = 30, mean age 41 years, range 18–63, seven women). PBMCs were obtained from healthy blood donors at the Department of Immunology and Transfusion Medicine, Oslo University Hospital, Oslo, Norway. All patients gave their written informed consent, and the study was approved by the Norwegian Regional Committee for Medical Research Ethics.

### Flow cytometric analysis

Nasal mucosal T cells were obtained as described [22]. Briefly, surgical specimens were finely minced with scissors and incubated with 1 mg/ml Liberase TM (Roche, Mannheim, Germany). Identically treated PBMCs served as controls for the collagenase-sensitivity of epitopes. To assess the cytokine production potential of mucosal T cells, dispersed cells were cultured for 4 h in RPMI/10% fetal calf serum (FCS) with 1.5 ng/ml phorbol 12-myristate 13-acetate (PMA) and 1 μg/ml ionomycin, with 10 μg/ml Brefeldin A (all from Sigma-Aldrich, St Louis, Mo) added after 1 h of stimulation to allow intracellular accumulation of cytokines. To detect IL-10, Golgi-Stop (BD Bioscience, San Jose, CA) was applied. Cells were stained with Fixable Viability Dye eFluor 450 (1 μl/10^6^ cells, eBioscience, San Diego, CA) for 30 min at 4°C, followed by surface staining with antibodies to CD3 (clone SK7), CD4 (clone OKT4), CD8 (clone SK1) and LAG3 (clone 3DS223H) from eBioscience, CD49b (clone P1E6-C5) from Biolegend (San Diego, CA), or CD45RO (clone UCHL-1) and CD127 (clone M21) from BD Biosciences. To detect intracellular cytokines, cells were treated with FOXP3/transcription factor staining buffer set according to the manufacturers protocol (eBioscience) and stained with antibodies to FOXP3 (clone 236A/E7), Helios (clone 22F6), and IL-17 (clone 64DEC) from eBioscience, or CTLA-4 (clone L3DTO), IFN-μ (clone 4S.B3), IL-2 (clone 17H12) and IL-10 (clone JES3-9D7) from Biolegend. All antibodies were incubated for 30 min at 4°C. Flow cytometry was performed on a BD LSRFortessa (BD Biosciences), and analyzed using FlowJo 7.6.3 software (Three Star, Eugene, OR). Gates for surface markers were set based on irrelevant isotype-matched antibodies, and cytokines gates was based on untreated cells.

### Statistics

P-values were calculated using paired and unpaired t-tests with the Graphpad Prism 5.0 (GraphPad Software, La Jolla, CA). A p-value of < 0.05 was considered significant.

## Results

### Phenotypic characteristics of FOXP3+ CD4+ T cells:

**Fig 1:**
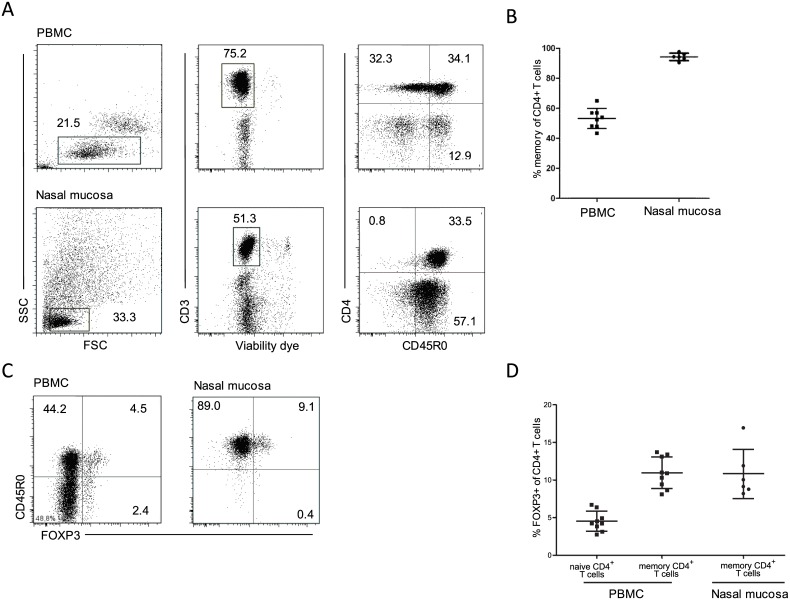
FOXP3+ CD4+ T cells in the steady-state nasal mucosa display a memory phenotype. (A) Samples of enzymatically digested nasal mucosa contained readily identifiable and viable CD3+ T cells. Representative plots showing the gating strategy when comparing the CD45RO-expression on CD4+ T cells derived from PBMCs or released from nasal mucosal tissue samples. (B) Percentage of CD4+ T cells expressing the memory marker CD45RO derived from PBMC (n = 9) or nasal mucosa (n = 7). (C) Representative plots comparing FOXP3-staining in CD4+ T cells derived from PBMCs or nasal mucosa. (D) Percentage of CD4+ T cells expressing FOXP3 in PBMC (n = 9) and nasal mucosa (n = 7).

To identify and characterize CD4+ T cells with regulatory functions in the nasal mucosa, we examined by flow cytometry single cell suspensions of enzymatically-digested nasal mucosa biopsies obtained from patients with septum deviation, but otherwise healthy. Peripheral blood obtained from healthy individuals was examined in parallel. By gating on viable cells we found that a mean of 95% of CD4+ T cells in the nasal mucosa expressed the memory marker CD45RO, whereas 54% of CD4+ T cells were CD45RO-positive in peripheral blood (Fig 1A and 1B).

**Fig 2:**
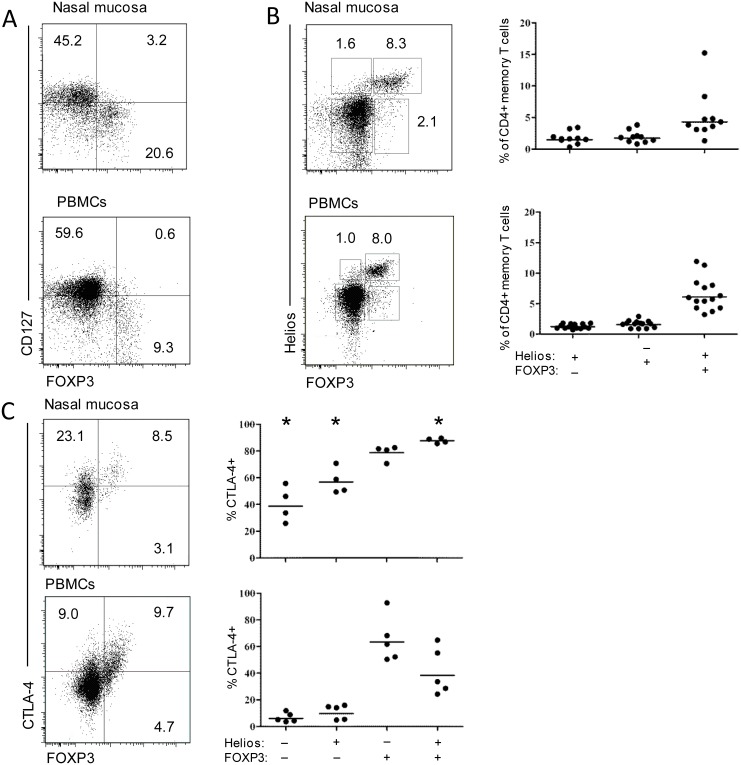
The majority of mucosal CD127lo FOXP3+ Helios+ CD4+ T cells express CTLA-4. (A) Representative plots gated on CD4+ T cells showing the expression of CD127 on FOXP3+ CD4+ T cells derived from nasal mucosa and in PBMC. (B) Representative plots (left) and compiled percentages of CD4+ T cells expressing FOXP3 and Helios in PBMC (n = 13) and in nasal mucosa (n = 11). (C) Representative plots (left) and percentages of intracellular CTLA-4 expression in CD4+ T cells subsets separated on FOXP3 and Helios expression in PBMC n = 5 and nasal mucosa (n = 4). Statistical difference between PMBC and nasal mucosa is indicated, *p<0.05.

Importantly, the finding that almost all CD4+ T cells in the nasal mucosa expressed CD45RO, excluded the possibility that the digested tissue was contaminated by cells from peripheral blood. A mean of 10% of nasal mucosa CD4+ T cells expressed the transcription factor FOXP3 (Fig 1C and 1D). In peripheral blood, 11% of CD4+CD45R0+ T cells expressed FOXP3, whereas 4% of naïve (CD45RO-) CD4+ T cells were FOXP3-positive (Fig 1C and 1D). Similar to FOXP3+ Tregs in peripheral blood [4], we found that the vast majority of FOXP3+CD4+ T cells in the nasal mucosa were CD127-negative (Fig 2A).

Preliminary experiments revealed that CD25 was cleaved off by the enzyme treatment, and we were thus unable to assess CD25 expression on tissue-derived cells by flow cytometry. However, we have previously shown by in situ staining of nasal mucosa specimens that most FOXP3+ T cells express high levels of CD25 [23]. Recently, the transcription factor Helios has been shown to distinguish between tTreg and pTregs, being preferentially expressed on the former [9]. We found that the majority (mean 70%) of FOXP3+ CD4+ T cells in the nasal mucosa expressed Helios (Fig 2B), which was similar to that found in peripheral blood (Fig 2B and [9]). In addition, we also found a small population of Helios-expressing CD4+ T cells that were negative for FOXP3 both in the nasal mucosa and peripheral blood (Fig 2B).

CTLA-4 is a potent negative regulator of T-cell immune responses, and has been shown to play a key role in the suppressive function by Tregs [24]. By intracellular staining we found that most (mean 87%) Helios+FOXP3+ T cells expressed CTLA-4, which was significantly higher (p = 0.015) than their counterparts in peripheral blood (Fig 2C). The majority of FOXP3+Helios- T cells both in the nasal mucosa and peripheral blood also co-expressed CTLA-4 (Fig 2C). FOXP3- CD4+ T cells in the nasal mucosa expressed lower levels of CTLA-4 than their FOXP3+ counterparts, but the levels were significantly higher (p = 0.015) compared with FOXP3- memory CD4+ T cells in peripheral blood (Fig 2C).

### Production of Th1, Th17 and Th2 cytokines by CD4+ T cells

**Fig 3:**
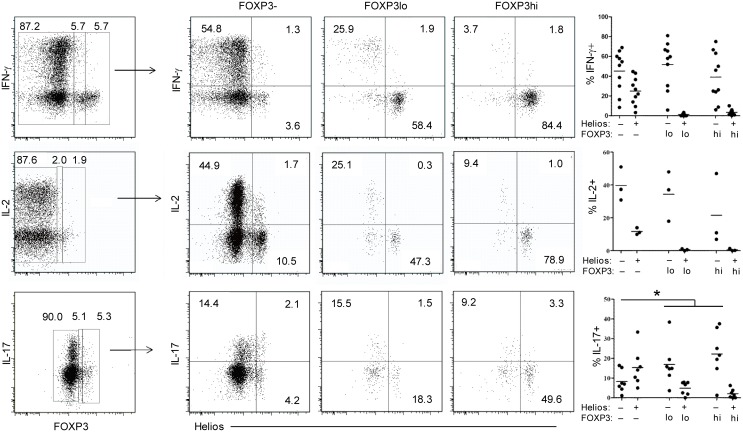
FOXP3hi and FOXP3lo CD4+ mucosal T-cells co-expressing Helios scarcely produce cytokines. Representative plots of nasal mucosal CD4+ T cells stained for IFN-μ (top), IL-2 (middle) and IL-17 (bottom) together with FOXP3 and Helios following 4 h stimulation with PMA/ionomycin in the presence of secretion blockade. FOXP3hi and FOXP3lo cells were defined by dividing the total proportion of FOXP3+ cells equally into a high- and low-intensity fraction. Compiled percentages (right panels) of cells expressing either IFN-μ (n = 10), IL-2 (n = 3) or IL-17 (n = 7). *p<0.03.

A characteristic property of FOXP3+ Treg-cell function is their reduced capacity to produce inflammatory cytokines [4,12–15]. To examine the ability of nasal mucosa-derived CD4+ T cells to produce cytokines, we stimulated tissue digests with PMA/ionomycin for 4 hours and assessed their intracellular cytokine expression by flow cytometry. It has been shown that the expression level of FOXP3 is associated with their suppressive capacity and their ability to produce cytokines [4,11], thus we divided the FOXP3+CD4+ T-cell population into FOXP3hi and FOXP3lo cells, based on dividing the total proportion of FOXP3+ cells equally into a high- and low-intensity fraction, followed by separation based on Helios expression. Strikingly, very few FOXP3+Helios+ CD4+ T cells produced either IFN-μ, IL-2, or IL-17, neither within the FOXP3hi and FOXP3lo fraction, whereas a large fraction of FOXP3+Helios-CD4+ T cells (both FOXP3hi and FOXP3lo cells) were cytokine-producing cells (Fig 3).

In fact, the fraction of FOXP3+Helios- CD4+ T cells expressing IL-17 were significantly higher (p = 0.03) than in the FOXP3-Helios- CD4+ T-cell subset, whereas the percentages of cells producing either IFN-μ or IL-2 were similar (Fig 3). We also measured the Th2 cytokines IL-4 and IL-5, however, only very few CD4+ T cells expressed these cytokines[25] and none of these co-expressed FOXP3 (not shown). The expression of GARP has been shown to selectively identify human FOXP3+ Tregs [26]. In agreement, we detected a subset of blood-derived T cells expressing GARP (not shown), however this molecule was cleaved off by enzyme treatment and therefore not useful as a marker for Tregs in human tissue (not shown).

Taken together, we find that both FOXP3hi and FOXP3lo CD4+ T-cells co-expressing Helios scarcely produced cytokines, which is similar to activated Tregs in peripheral blood [4]. In contrast, FOXP3+Helios- T cells appeared to be a heterogeneous population of cells containing many cells producing either Th1 and/or Th17 cytokines.

### Identification of IL-10-producing CD4+ T cells

**Fig 4:**
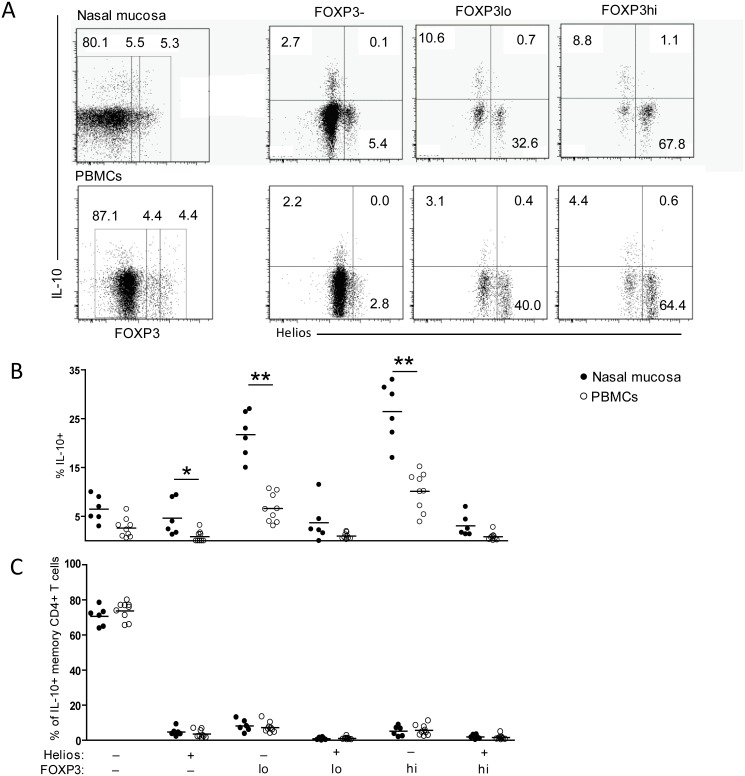
IL-10 production in mucosal FOXP3+ CD4+ T cells is most prominent in cells lacking Helios. (A) Representative plots of gating strategy of CD4+ T cells from nasal mucosa and PBMCs when comparing IL-10 and Helios staining with increasing levels of FOXP3 expression, revealing that IL-10 is preferentially expressed by FOXP3+ cells negative for Helios. (B) Compiled percentages from mucosal samples (n = 6) and PBMC (n = 9) Statistical difference between PBMC and nasal mucosa is indicated, *p<0.01, **p<0.0004. (C) Compiled percentages of IL-10+ memory CD4+ T cell distribution within these subsets from nasal mucosa samples (n = 6) and PBMC (n = 9) reveal that the vast majority of IL-10+ cells are found within the FOXP3-Helios- population.

A characteristic of many CD4+ T cells with regulatory properties is their capacity to produce the immunosuppressive cytokine IL-10. We thus examined the ability of nasal mucosa-derived CD4+ T cells to produce IL-10 in response to PMA/ionomycin stimulation. CD4+ T cells from peripheral blood obtained from healthy individuals were examined in parallel. By intracellular staining, we found that only a few mucosal FOXP3+Helios+ T cells produced IL-10 (mean 2%, Fig 4A and 4B). There was no difference between cells expressing high or low levels of FOXP3 (Fig 4A and 4B).

**Fig 5:**
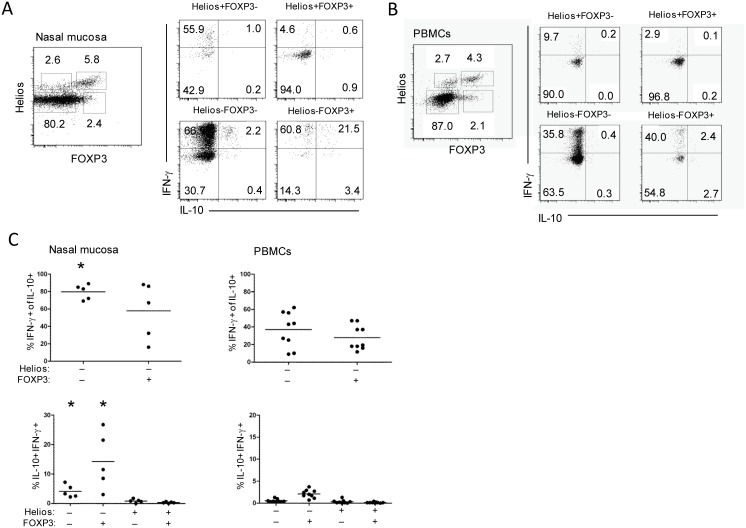
IL-10 is preferentially coexpressed with IFN-μ by mucosal CD4+ T cells. Representative plots showing the gating strategy of CD4+ T cells from nasal mucosa (A) and PBMCs (B) when comparing production of IL-10 and IFN-μ in cells expressing FOXP3 and Helios. (C) Compiled percentages from mucosal samples (n = 5) and PBMC (n = 9). Statistical difference between nasal mucosa and PBMC is indicated, *p<0.05.

In contrast, among FOXP3+Helios- CD4+T cells (both FOXP3 hi and low cells) a large fraction were IL-10-producing (mean 24%), which was significantly higher (p = 0.0004) than their counterparts in peripheral blood (Fig 4A and 4B). Also, nasal mucosa-derived FOXP3- CD4+ T cells contained significantly more (p = 0.01) IL-10-producing cells than their peripheral blood counterparts (Fig 4A and 4B). However, analysis of the distribution of IL-10+ memory CD4+ T cell within these subsets from mucosa samples and PBMC revealed that the vast majority of IL-10+ cells (mean 70% and 73%, respectively) are found within the FOXP3-Helios- population (Fig 4C). Although we found that FOXP3+Helios- T cells contained the highest fraction of IL-10-producing cells, this subset also contained many cells that produced IFN-μ and IL-17 (Fig 3). To assess whether the IL-10-producing cells co-expressed any of these inflammatory cytokines, we co-stained for IL-10 together with IFN-μ or IL-17. This revealed that a substantial fraction (mean 57%) of mucosal IL-10+FOXP3+Helios- CD4+ T cells co-expressed IFN-μ (Fig 5A and 5C), and the percentage of IL-10+ IFN-μ+ CD4+ T cells was significantly higher (p = 0.0008) than in peripheral blood (Fig 5B and 5C).

Only a minor fraction of IL-10+FOXP3+Helios- CD4+ T cells co-produced IL-17 (data not shown).

We also found that 6% of FOXP3-Helios- CD4+ T cells in the nasal mucosa produced IL-10 (Fig 4A), and the majority of these (mean 79%) co-expressed IFN-μ, which was significantly higher than observed in peripheral blood (p = 0.0004, Fig 5A and 5C). Moreover, the percentage of IL-10+ IFN-μ+ FOXP3- CD4+ T cells was also significantly higher (p = 0.0004) than in blood (Fig 5A and 5C). This phenotype is compatible with Tr1 cells, which are characterized by their production of IL-10, often concomitant with IFN-μ [16]. It has been shown that Tr1 cells can be identified in peripheral blood by their co-expression of CD49d and LAG-3 [16]. We detected this T cell phenotype (CD49d+LAG-3+CD4+CD3+) in peripheral blood, however, using the same antibody combination we were unable to detect any CD4+ T cells expressing LAG-3 in nasal mucosa-derived cell suspensions (S1 Fig). This was not dependent on the enzymatic treatment as LAG-3 expression on blood T cells was unaffected by collagenase treatment (not shown).

## Discussion

To maintain immune homeostasis in the upper airway mucosa, immune effector functions must be under strict regulatory control to avoid unwanted or excessive immune reactions. Moreover, accumulating evidence suggests that Tregs play a crucial role in maintaining homeostasis in peripheral tissues, but studies directly examining Tregs at mucosal surfaces of humans are limited.

Similar to that shown in peripheral blood ([9] and in this study)) we found that 70% of FOXP3+CD4+ T cells in the nasal mucosa were Helios+CD45RO+CTLA4+CD127-. We were not able to determine their CD25 expression because of epitope cleavage by the collagenase treatment, but previous work by our group has shown that most FOXP3+ T cells in the nasal mucosa also express high levels of CD25 [23]. Moreover, FOXP3+Helios+ CD4+ T cells scarcely produced any of the cytokines analyzed (IFN-μ, IL-2, IL-4, IL-5, IL-10, and IL-17). Together, these properties corresponded well with that recently reported for Helios+ Tregs in peripheral blood [12–15] and activated Tregs as described by Miyara et al. [4], which in all studies were shown to be highly suppressive cells in vitro. Furthermore, in the latter study it was shown that only FOXP3hi cells exhibited suppressive activity, whereas FOXP3lo T cells were unable to suppress and produced high levels of IL-2, IFN-μ and IL-17. However, using Helios as an additional marker, we found that all nasal mucosa-derived FOXP3+CD4+ T cells (both FOXP3hi and lo) co-expressing Helios, scarcely produced cytokines. This finding is in agreement with Raffin et al. showing that peripheral blood FOXP3+Helios+CD4+ T cells produced low levels of cytokines and displayed high suppressive activity [12]. Expression of Helios by FOXP3+ T cells has been suggested to specifically identify tTregs [8]. This notion has later been questioned because it was shown that activated T cells transiently express both FOXP3 and Helios [27]. However, the finding that FOXP3+Helios+CD4+CD127-CTLA4+CD45RO+ T cells hardly produced any cytokines in response to a strong polyclonal stimulus, strongly suggested that we have identified a population of tTregs in the nasal mucosa, which constitute ~7% of all resident CD4+ T cells under steady-state conditions. This is also in agreement with a recent study demonstrating that Helios was required to keep a stable inhibitory activity of Tregs, and that mouse Tregs lacking Helios produced increased amounts of proinflammatory cytokines during inflammation [11]. In humans, the expression of GARP has been shown to selectively identify FOXP3+ Tregs [26]. However, this surface protein was sensitive to enzyme treatment and therefore not possible to assess in enzymatically digested tissue. Moreover, unfortunately, due to the limited number of T cells that can be isolated from nasal mucosa biopsies, we were not able to perform suppression experiments ex vivo.

The Helios-negative fraction of FOXP3+CTLA4+CD45RO+CD127-CD4+ T cells, constituting ~3% of nasal mucosa CD4+ T cells, were heterogeneous in terms of cytokine production. Importantly, in terms of regulatory function, FOXP3+Helios- CD4+ T cells contained a significantly higher fraction of IL-10-producing cells compared to FOXP3- T cells in the nasal mucosa as well as their FOXP3+Helios- CD4+ T cell counterparts in peripheral blood (Fig 4). It has been shown in experimental mice that disruption of IL-10 production selectively in FoxP3+ CD4+ T cells led to immune-mediated inflammation in the gut, skin and lung [3]. In contrast, IL-10 production by FoxP3+ T cells was not required for the control of systemic autoimmunity. This suggested that production of IL-10 by FoxP3+ Tregs may have a non-redundant role for the protection of unwanted immune reactions to foreign antigens at mucosal surfaces. The finding that IL-10-producing FOXP3+ Tregs are more frequent at mucosal surfaces than in other tissues and blood (as shown here) further suggests that these cells are particularly important at sites with high antigenic exposure. We found that approximately 50% of IL-10+FOXP3+ T cells co-produced IFN-μ. Interestingly, CD4+ T cells with the same phenotype were shown to inhibit the development of hyper-reactivity in an mouse model for asthma [28]. It has also been shown that T cells producing IL-10- and IFN-μ increase in numbers during immunotherapy in allergic rhinitis patients [29].

FOXP3+Helios- T cells also contained the highest percentage of IL-17-producing cells. In fact, this subset contained twice as many IL-17+ T cells as compared with FOXP3- CD4+ T cells. Moreover, a substantial fraction of these FOXP3+ T cells co-expressed IL-2 and IFN-μ. A similar phenotype has been reported for FOXP3+Helios- CD4+ T cells in peripheral blood [4,12,15]. However, whereas these cytokine-producing FOXP3+CD4+ T cells in some studies were shown to be suppressive ex vivo [12], others have shown that such cells were non-suppressive [4]. We found that only a minor fraction of nasal mucosa-derived FOXP3+ T cells co-produced IL-10 and IL-17, which may suggest that these are functionally distinct subpopulations. Taken together, these findings indicate that FOXP3+Helios- T cells are heterogeneous in term of function and may contain several partly overlapping subsets, including T cells with both effector and suppressive functions.

Similar numbers of FOXP3+ Tregs with regulatory activity have been found in the human large intestine [30]. A fraction of these, albeit lower than in the nasal mucosa, co-express Helios. Moreover, similar to the upper airways, virtually all FOXP3+ Tregs in the large intestine express CTLA-4. This suggests that CTLA-4 may play an important suppressive role at both mucosal sites by cross-linking B7 molecules on activated antigen presenting cells and T cells [31]. However, it is apparent from studies in mice that the function of Foxp3+ Tregs in the gut depends on tissue-specific factors [21]. Therefore, whether such tissue-specific factors are important for Treg functions in the upper airways needs to be determined.

As reported earlier, we found that the immunomodulatory cytokine IL-10 also is produced by FOXP3-CD4+ T cells. In fact, we show here that in terms of absolute numbers, most IL-10-producing CD4+ T cells in the nasal mucosa are found within the FOXP3- CD4+ T-cell subset, and more than 5% of all FOXP3- CD4+ T cells produced IL-10 in response to PMA/ionomycin, which was significantly higher than in peripheral blood (Fig 4). Moreover, 80% of these CD4+ T cells co-produced IFN-μ, whereas less than 40% of FOXP3-CD4+ T cells co-expressed IFN-μ in blood. The ability of effector CD4+ T cells to co-express IL-10 and IFN-μ is well established, and such cells have been described in several infection models [32,33]. In fact, IL-10+ IFN-μ+ FOXP3-CD4+ T cells are believed to prevent collateral damage through its effect by IL-10, but may also be exploited by the microbes to prevent elimination [34]. Moreover, non-exposed mice display few IL-10+ IFNγ+ T cells, suggesting that repeated antigen-stimulation is needed for their maintenance.

Tr1 cells have been shown to promote and maintain tolerance in the periphery, and secrete high amounts of IL-10, often concomitant with high levels of IFN-μ. It was recently shown that Tr1 cells could be identified in blood by their co-expression of CD49d and LAG-3 [16]. Applying the same antibody combination we were not able to find any CD49d+LAG-3+ T cells in the nasal mucosa. This finding either suggested that Tr1 cells do not occur in the upper airways during the steady-state or that this T-cell subset change their phenotype in the tissue. Whether the expression of LAG-3 on Tr1 cells is functionally important has not been determined, but it was recently shown that IL-27 induced expression of LAG-3 on Foxp3+ Treg cells had suppressive functions in a colitis model in mice [35].

Interestingly, CD4+ T cells in the nasal mucosa displayed several characteristics that differed from their counterparts in peripheral blood. FOXP3+Helios+ T cells in the nasal mucosa expressed higher levels of CTLA-4 and both FOXP3+ and FOXP3- T cells expressed significantly more IL-10. Higher numbers of IL-10+ IFN-μ+ T cells were also found in the mucosa. This indicates that the local microenvironment in the upper airways shape the T-cell compartment either by creating a survival niche for selected T cells or by changing their functional properties after entering the tissue. Either way, our results show the importance of studying T cells directly in the environment were the normally reside.

The IL-10 receptor is expressed on many cell types and IL-10 has a broad range of anti-inflammatory activities. Interestingly, in a clinical study it was found that IL-10 receptor-deficient patients suffered from both an early onset life-threatening enterocolitis and recurrent upper airway infections [36]. These findings indicate that IL-10 plays an important role in immunological homeostasis of upper airway mucosa, including limiting tissue damage associated with an immune response to airway pathogens.

Taken together, based on phenotypic and functional characteristics, we find that the nasal mucosa contains several distinct subsets of CD4+ T cells with regulatory functions. In keeping with previous reports, our findings strongly suggest that the majority of FOXP3+ Tregs, being Helios+CTLA4+CD45RO+ cells producing low levels of cytokines, are thymus-derived Treg cells. Conversely, FOXP3+ T cells lacking Helios produced multiple cytokines, suggesting that this population is heterogeneous in terms of function. Moreover, a large fraction of both FOXP3+ and FOXP3- CD4+ T cells expressed IL-10, further strengthening the notion that IL-10 is an important immunosuppressive cytokine at mucosal surfaces [3]. Manipulation of Tregs has been suggested as an attractive approach to treat allergic diseases, such as allergic rhinitis. However, we show here that several distinct subsets of CD4+ T cells with regulatory functions reside in the nasal mucosa during steady state. These subsets are most likely co-existing in a tightly controlled equilibrium which should be taken into account when targeting Tregs for therapy.

## Supporting Information

S1 Fig Analysis of co-expression of CD49b and LAG-3 on CD4+ T cells in PBMCs and dispersed from nasal mucosa, revealing lack of LAG-3-expression on mucosal T cells. Representative dot-plots from 3 independent experiments.
(EPS)
